# Multi-Objective Optimal Allocation of River Basin Water Resources under Full Probability Scenarios Considering Wet–Dry Encounters: A Case Study of Yellow River Basin

**DOI:** 10.3390/ijerph182111652

**Published:** 2021-11-06

**Authors:** Xike Guan, Zengchuan Dong, Yun Luo, Dunyu Zhong

**Affiliations:** College of Hydrology and Water Resources, Hohai University, Nanjing 210098, China; xike_guan@126.com (X.G.); luoyun_hhu@hhu.edu.cn (Y.L.); hhuzhongdy@163.com (D.Z.)

**Keywords:** wet–dry encounter, copula, multi-objective allocation model, water resources, Yellow River Basin

## Abstract

Wet–dry encounters between basins and regions have an important impact on the allocation of water resources. This study proposes a multi-objective allocation model for basin water resources under full probability scenarios considering wet–dry encounters (FPS-MOWAM) to solve the problem of basin water resource allocation. In the FPS-MOWAM model, the sub-regions were merged by precipitation correlation analysis. Next, the joint probability distribution of basin runoff and region precipitation was constructed using copula functions. The possible wet–dry encounter scenarios and their probabilities were then acquired. Finally, the multi-objective allocation model of water resources was constructed using the full probability scenario for wet–dry encounters in each region. The FPS-MOWAM is calculated by the NSGA-II algorithm and the optimal water resource allocation scheme was selected using the fuzzy comprehensive evaluation method. Using the Yellow River Basin as an example, the following conclusions were obtained: (1) the Yellow River Basin can be divided into four sub-regions based on precipitation correlations: Qh-Sc (Qinghai, Sichuan), Sg-Nx-Nmg (Gansu, Ningxia, Inner Mongolia), Sxq-Sxj (Shaanxi, Shanxi), and Hn-Sd (Henan, Shandong), (2) the inconsistencies in synchronous–asynchronous encounter probabilities in the Yellow River Basin were significant (the asynchronous probabilities were 0.763), whereas the asynchronous probabilities among the four regions were 0.632, 0.932, and 0.763 under the high, medium, and low flow conditions in the Yellow River Basin respectively, and (3) the allocation of water resources tends to increase with time, allocating the most during dry years. In 2035, the expected economic benefits are between 11,982.7 billion CNY and 12,499.6 billion CNY, while the expected water shortage rate is between 2.02% and 3.43%. In 2050, the expected economic benefits are between 21,291.4 billion CNY and 21,781.3 billion CNY, while the expected water shortage rate is between 1.28% and 6.05%.

## 1. Introduction

Water resources, as strategic natural and public social resources, are directly related to the interests of the state and its prosperity, people, and related social development [1,2]. As socioeconomic development has sped up, the demand for water resources has increased sharply, with a contradiction gradually appearing between supply and demand thereof, while an uneven spatiotemporal distribution of precipitation has exacerbated the shortage of water resources in some areas [3,4,5]. The question of how to allocate water resources scientifically, reasonably, and fairly, while ensuring that limited water resources produce greater economic benefits and promoting sustainable socioeconomic and ecosystem development, has attracted the attention of governments and scholars around the world [6,7,8].

Water allocation is an important tool for water resource management. Ever since Masse [9] (1946) proposed the reservoir optimal operation problem, the water resource allocation problem has been widely studied by an increasing number of scholars [10,11]. Given this in-depth study, water resource allocation strategies have developed from a single goal of being applicable to a single water sector to a multi-regional, multi-sector, and multi-objective integrated system [12,13,14,15]. The methods to find the optimal allocation of water resources have developed from linear programming [16] and dynamic programming [17] to multi-objective [18] and group decision-making [19]. With the development of applied mathematics, game theory [20,21], fuzzy mathematics theory [22], and system science theory [23] have also been applied to address the problem. Furthermore, developments in modern bionics algorithms, genetic algorithms [24,25], neural networks [26,27], and simulated annealing algorithms [28] have also been gradually applied to studying water resource allocation.

The aim of water allocation is to improve the efficiency of water resource use through equitable allocation of water resources and to achieve a rational, regulated use of water resources [29]. Water allocation requires social, economic, and environmental decision-making objectives. In previous studies, the optimal water allocation solution is usually sought for a particular scenario, using a multi-year average or using the same frequency of basins and regions [30]. In practice, large basins often have inconsistencies between the basin and region’s wet–dry encounters, presenting a significant challenge to basin water allocation [31,32]. Traditional water resource allocation does not effectively respond to the needs of water allocation under the wet–dry encounter scenario, which affects the efficacy and implementation of water resource allocation. The impacts of the wet–dry encounter on water resource allocation include: (1) increased complexity of water resource allocation in consistent and inconsistent abundance and depletion, and (2) increased difficulty of making decisions regarding water allocation options. The study of water resource allocation in basins under the influence of wet–dry encounters can make the allocation scheme more suitable for the water demand in the basin and is paramount for the fine-grained allocation of water resources.

The wet–dry encounters in basin water allocation are a combination of encounters of multidimensional hydrological variables [33,34]. The encounter problem of hydrological variables is usually solved using a multivariate joint distribution function, which can be divided into multivariate joint distributions with the same or different marginal distributions. According to the characteristics of hydrological variables in water resource allocation, precipitation and runoff often have different marginal distributions. Therefore, using a different marginal distribution function for each variable is more appropriate. The copula function has a flexible structure that allows for the construction of joint distribution functions with any marginal distribution and has been widely studied for solving multivariate inter-relationships [35,36]. In hydrology, two-dimensional or three-dimensional copula functions are commonly used to establish functional relationships for binary or ternary hydrological variables [37,38,39]. However, more complex situations are often encountered in practical applications where two or three dimensions do not accurately reflect the actual requirements, and multi-dimensional copula functions need to be established to better suit the actual needs.

**Figure 1:**
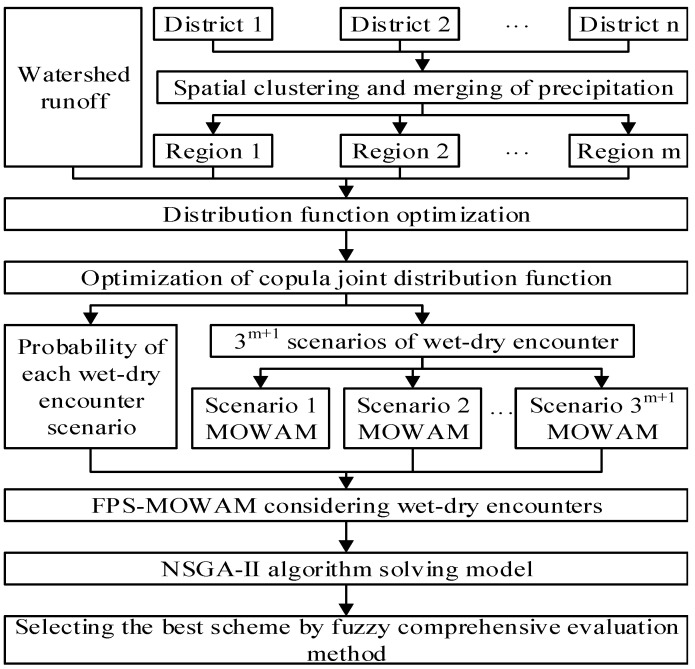
The flow diagram for the full probability scenarios considering wet–dry encounters between basins and regions (FPS-MOWAM).

The goal of this study was to develop a multi-objective optimal allocation model of water resources in the full probability scenarios considering wet–dry encounters between basins and regions (FPS-MOWAM), utilizing the copula function to examine water resource allocation in a basin under wet–dry encounter scenarios. The objectives were: (1) to analyse the basin–region wet–dry encounter scenarios and probabilities based on the joint distribution function using copula functions for basin runoff and regional precipitation, and (2) to construct a multi-objective optimal allocation model for water resources considering the encounter. On the one hand, the model is more comprehensive than traditional allocation models in setting water resource allocation scenarios, rather than only studying partial scenarios. On the other hand, the allocation scenarios are better adapted to the actual needs and the ability to cope with various climate changes is enhanced. The research process is illustrated in Figure 1.

## 2. Study Area and Data Source

### 2.1. Study Area

The Yellow River (Yr) is one of the largest rivers in the world, with a basin area of 795,000 km^2^, an origin in the Tanggula Mountains, and a course that flows through Qinghai (Qh), Sichuan (Sc), Gansu (Gs), Ningxia (Nx), Inner Mongolia (Nmg), Shaanxi (Sxq), Shanxi (Sxj), Henan (Hn), Shandong (Sd), and into the Bohai Sea at Lijin, Shandong. The climate of the basin is continental and there is a large inter-annual variation in precipitation. The average annual precipitation in the basin is 446 mm and the precipitation trend decreases from southeast to northwest. The annual average water resources provided by the Yellow River is 71.94 billion m^3^, amounting to 473 m^3^ per capita, and this is significantly lower than the international standard of 1000 m^3^ per capita in areas considered to be experiencing water shortages.

In the past 40 years, the rapid socioeconomic development in the Yellow River Basin has meant that the demand for water resources in each province has increased sharply and the consequent water shortage problem has become increasingly serious. The current water distribution scheme for the Yellow River Basin was issued by the State Council in 1987 (87WRAP) [40]. This scheme is based on the socioeconomic development and industrial layout from that time, and the water demand for the provinces in 2000 was also predicted according to the trend in development from that time. The runoff from the Yellow River Basin under the multi-year average is distributed among the provinces. However, due to the uneven development of each province, there were some differences between the actual water demand and the results predicted in the 87WRAP; for example, the actual water intake in Shandong exceeded the proportion of water diverted, while the actual water intake in Shanxi and Shaanxi has not yet reached the allocated level. Therefore, making appropriate adjustments to the water distribution in the Yellow River Basin to meet the water resource needs in each province under the current conditions is a step that must be urgently taken. However, the 87WRAP distributed water according to annual average runoff in the same proportion, that is, ignoring the wet–dry encounters between the Yellow River Basin and the provinces. This further aggravates the conflict between provinces caused by water resource distribution problems in areas of water scarcity.

Water supply parameters in the water resource allocation model include the groundwater, external water, and surface water supplies. The groundwater supply uses the average annual amount of recoverable groundwater for local use only. The external water supply includes the water supply for specific provinces, which is only for local use, and the water supply for the entire basin that is involved in the overall water resource allocation for the area. The typical year method is used to select representative years from a historical sequence as the input terms for the surface water runoff levels used in the model. Using runoff data from 1960 to 2010, 1982 was selected as the representative high flow year, 2007 as the representative medium flow year, and 2004 as the representative low flow year. Based on the two 15-year planning requirements proposed by the Chinese government, 2035 and 2050 were selected as the planning years for water resource allocation. The water demand from each province in the Yellow River Basin in 2035 and 2050 was predicted using the quota method [41].

**Figure 2:**
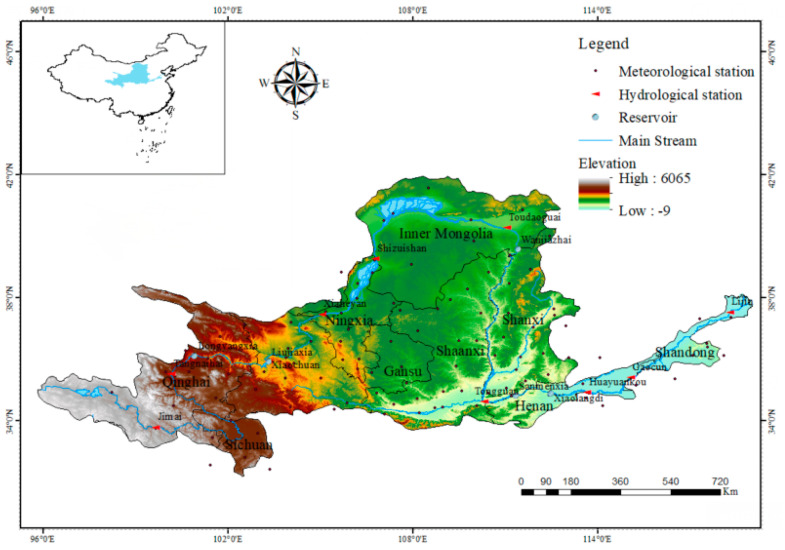
The geographical location of the Yellow River Basin, provinces, and hydrological and meteorological stations from where data were obtained for this study.

To analyse the wet–dry encounters between basins and provinces, the runoff recorded at the Lijin hydrological station was used to reflect the high, medium, and low flows in the Yellow River Basin, and the precipitation data were used to reflect the high, medium, and low flow conditions for each province. The geographical locations of the Yellow River Basin, provinces, hydrological stations, and meteorological stations are shown in Figure 2.

### 2.2. Data Sources

Water resources and socioeconomic data were sourced from the Yellow River Basin Water Resources Bulletin (http://www.yrcc.gov.cn/zwzc/gzgb/gb/szygb/) (accessed on 3 November 2021) and the China Statistical Yearbook (http://www.stats.gov.cn/tjsj/ndsj/) (accessed on 3 November 2021). Daily precipitation data from 107 national meteorological stations in the Yellow River Basin (for the period 1960–2010) were sourced from the China Meteorological Data Network (http://data.cma.cn/) (accessed on 3 November 2021).

## 3. Research Methodology

### 3.1. Scenario Setting

The marginal distribution function of each hydrological variable was fitted with an appropriate distribution function based on a historical time series. Then, several percentiles were selected—based on a marginal distribution function—to divide the variables into dry and wet years. In China, 37.5% and 62.5% are usually selected as the percentages of dry and wet years, and the hydrological variables are divided into three levels: high, medium, and low.

The annual runoff and regional average annual precipitation were used to analyse wet–dry encounters between basins and regions. Assuming that the basin is divided into m regions, there will be 3^m^ scenarios of wet–dry encounters among the regions and 3^m+1^ scenarios of wet–dry encounters between basins and regions, including all wet–dry encounter scenarios.

### 3.2. Analysis of Wet–Dry Encounters between Basins and Regions

The copula is a function proposed by Sklar [42] to capture the joint distribution of two or more random variables and has a marginal distribution of unlimited variables. It can connect *n* arbitrary marginal distributions to generate a multivariable joint distribution function. The Archimedean copula function has an explicit analytical formula and generator expression that has been widely used in hydrology [43]. The expression of the n-dimensional Archimedean copula function can be written as follows:

(1) $$C^{n}\left(u_{1},\cdots ,u_{n}\right)=\phi^{-1}\left[\phi \left(u_{1}\right)+\cdots +\phi \left(u_{n}\right)\right]$$

where $C^{n}\left(u_{1},\cdots ,u_{n}\right)$ is a copula function that reflects the structure of correlations among *n* random variables, $u_{1},\cdots ,u_{n}$ are cumulative distribution functions of *n* random variables respectively, ϕ is an Archimedean generating function, which is continuous and strictly decreasing convex, and $\phi^{-1}$ is the inverse function of ϕ.

The steps of wet–dry encounters between basins and regions, which are based on the copula function, are as follows: (1) Spatial clustering and merging of precipitation information [44], in which a hierarchical cluster analysis method was used to merge regions according to their precipitation correlations. (2) Determining and selecting the appropriate marginal distribution from a variety thereof, and determining the marginal distribution with the best-fitting effect using the goodness-of-fit test. (3) Determining the appropriate copula function with the best-fitting effect using the goodness-of-fit test and, based on the characteristics of correlation between variables, selecting one of the former to fit to the joint distribution function and estimate the parameters. Finally, (4) analysing possible wet–dry encounter scenarios and their probabilities.

### 3.3. Full Probability Scenarios Considering Wet–Dry Encounters between Basins and Regions (FPS-MOWAM) Considering Wet–Dry Encounters

Water resource allocation schemes for basins with inconsistent wet–dry encounters are not well-adapted to the actual conditions therein under a single scenario or using conditions based on a basin and region with the same frequency of wet–dry encounters. Although many scholars have paid attention to the impact of the uncertainty of hydrological variables on water resource allocation, wet–dry encounters between basins and regions were not included among these.

This study proposes the FPS-MOWAM as a way to analyse the optimal allocation of water resources under different wet–dry encounter scenarios. In this model, the objective of a single scenario is to minimise the sum of the squares of water shortage rates and maximise economic benefits. In the full probability scenario, a single scenario is combined with the probability that it will occur to ensure that the water shortage rate is at the minimum expected and the economic benefits are at the maximum expected under all scenarios. There may be great differences in the scenarios and probabilities of wet–dry encounters in regions in wet, normal, and dry years in the basin. Therefore, to refine the water resource allocation scheme, the full probability scenario for wet–dry encounters in regions under the probability that wet, normal, and dry years would occur in the basin is set as the scenario for the allocation scheme.

#### 3.3.1. Objective Functions

##### Equality Objective

For the entire scenario scheme, the equality objective considers various possibilities and probabilities for wet–dry encounter scenarios. The expectation of the equality objective is then, when combined with the equality objective value in the allocation model under the condition of a single scenario, used in all scenarios as the objective of the full-scenario scheme, as follows:

(2) $$\min g_{1}=\sum_{s=1}^{S}p_{s}•f_{1s}$$

where $p_{s}$ denotes the probability of the scenario *s* and $f_{1s}$ denotes the equality objective value of scenario *s,* represented by the minimum sum of squares of water shortage rates in each region of the scenario, as below:

(3) $$\min f_{1s}=\sum_{i=1}^{I}{\left[\frac{D_{is}-\sum_{j=1}^{J}W_{ijs}}{D_{is}}\right]}^{2}$$

where $D_{is}$ denotes the water demand from region *i* under scenario *s* and $W_{ijs}$ denotes the supply from water source *j* in area *i* under scenario *s*.

##### Benefit Objective

The full-scenario benefit objectives also need to consider the values of the benefit objectives of all scenarios and the probability that they will occur using the expectations of the benefit objectives of all scenarios as the benefit objectives of the full-scenario programme, as shown below:

(4) $$\max g_{2}=\sum_{s=1}^{S}p_{s}•f_{2s}$$

where $f_{2s}$ denotes the benefit objective value of scenario *s* and is represented by the maximum economic benefit in each region of the scenario, as follows:

(5) $$\max f_{2s}=\sum_{i=1}^{I}\sum_{k=1}^{K}\left(b_{iks}\sum_{j=1}^{J}W_{ijk}-c_{iks}\sum_{j=1}^{J}W_{ijk}\right)$$

where $b_{ik}$ is the benefit from the water supply to water consumption department *k* in region *i* under scenario *s* and $c_{ik}$ is the cost of the water supply to water consumption department *k* in region *i* under scenario *s*.

#### 3.3.2. Constraint Setting

##### Constraints on Water Supply and Demand

Constraints on water supply and demand include those related to groundwater and river reach water supply capacity as well as total water supply and demand. Here, the groundwater supply did not exceed its maximum exploitable capacity and the rainwater supply did not exceed its maximum water supply. The water supply in the area did not exceed the expected water demand:

(6) $$W_{igs}\leq W_{igs,\max}$$

(7) $$Q_{ns}\leq Q_{ns,\max}$$

(8) $$\sum_{j=1}^{J}W_{ijs}\leq \sum_{s=1}^{S}p_{s}•D_{is}$$

where $W_{igs}$ is the groundwater supply in region *i* under scenario *s*, $W_{igs,\max}$ is the maximum exploitable groundwater in region *i* under scenario *s*, $Q_{ns}$ is the water supply in reach *n* under scenario *s*, and $Q_{ns,\max}$ is the maximum water supply in reach *n* under scenario *s*.

##### Constraints on Reservoir Water

Constraints on reservoir water include those related to storage capacity and water balance. The reservoir capacity constraint indicates that the reservoir capacity should be within a certain range at time *t*, and the water balance constraint requires the reservoir capacity to change with the inflow, outflow, and evaporation loss over a period of time:

(9) $$Z_{mst,\min}\leq Z_{mst}\leq Z_{mst,\max}$$

(10) $$V_{ms,t}-V_{ms,t-1}=(I_{ms,t}-Q_{ms,t}-E_{ms,t})\Delta t$$

where $Z_{mst}$ is the water level of reservoir *m* at time *t* under scenario *s*, $Z_{mst,\min},Z_{mst,\max}$ are the minimum and maximum water level of reservoir *m* at time *t* under scenario *s* respectively, $V_{ms,t},V_{ms,t-1}$ are the capacity of reservoir *m* at time *t* and *t*-1 under scenario *s* respectively, and $I_{ms,t},Q_{ms,t},E_{ms,t}$ are the inflow, outflow, and evaporation loss of reservoir *m* at time *t* under scenario *s*, respectively.

##### Constraints on Eco-Environmental Flow

Constraints on the eco-environmental flow include the minimum ecological flow, minimum sand flushing flow, and minimum anti-bulb flow. The maximum value of these three constraints is considered the constraint on the eco-environment flow:

(11) $$\max\left(q_{kst,e,\min},q_{kst,s,\min},q_{kst,ice,\min}\right)\leq q_{kst}$$

where $q_{kst}$ is the eco-environment flow of hydrological station *k* at time *t* under scenario *s* and $q_{kst,e,\min},q_{kst,s,\min},q_{kst,ice,\min}$ are, respectively, the minimum ecological flow, minimum sand flushing flow, and minimum anti-bulb flow of hydrological station *k* at time *t* under scenario *s*.

##### Constraints on Non-Negative Variables


(12) $$Q_{ikt}\geq 0,W_{ijkt}\geq 0,D_{it}\geq 0$$

#### 3.3.3. Global Model

The FPS-MOWAM model, which integrates the above objectives and constraints, can be described as follows:

(13) $$\left\{ \begin{array}{ll} \mathrm{Over}\;\mathrm{goal} & \text{Fair goal }:g_{1}=\min\sum_{s=1}^{S}p_{s}•f_{1s}\\ & \text{Benefit goal}:g_{2}=\max\sum_{s=1}^{S}p_{s}•f_{2s}\\ & \left\{ \begin{array}{ll} \mathrm{Single}\;\mathrm{scenario}\;\mathrm{goal}: & \text{Fair goal }:f_{1s}=\sum_{i=1}^{I}{\left[\frac{D_{is}-\sum_{j=1}^{J}W_{ijs}}{D_{is}}\right]}^{2}\\ & \text{Benefit goal}:f_{2s}=\sum_{i=1}^{I}\sum_{k=1}^{K}\left(b_{ik}\sum_{j=1}^{J}W_{ijks}-c_{ik}\sum_{j=1}^{J}W_{ijks}\right)\\ & s.t.\{\begin{array}{c} W_{igs}\leq W_{igs,\max}\\ Q_{ns}\leq Q_{ns,\max}\\ Z_{mst,\min}\leq Z_{mst}\leq Z_{mst,\max}\\ V_{ms,t}-V_{ms,t-1}=(I_{ms,t}-Q_{ms,t}-E_{ms,t})\Delta t\\ \max\left(q_{kst,e,\min},q_{kst,s,\min},q_{kst,ice,\min}\right)\leq q_{kst}\\ Q_{ikt}\geq 0,W_{ijkt}\geq 0,D_{it}\geq 0 \end{array} \end{array}\right.\\ & s.t.\begin{array}{cc} & \sum_{j=1}^{J}W_{ijs}\leq \sum_{s=1}^{S}p_{s}•D_{is} \end{array} \end{array}\right.$$

### 3.4. Model Solution and Scheme Optimisation

#### 3.4.1. Solution of Multi-Objective Model Based on NSGA-II

The NSGA-II algorithm was used to solve the FPS-MOWAM. The steps of the NSGA-II algorithm are as follows.

Step 1: Parameter selection. According to the research of Jia et al. [45] and Zhang et al. [3], the parameters in the NSGA-II algorithm set the population size (N) to 100, maximum number of iterations to 1000, crossover probability to 0.75, and mutation probability to 0.01, and these parameters, overall, produce excellent results from the model.

Step 2: Initialise the population. The initial population is generated randomly using randomly generated numbers.

Step 3: Fast non-dominated sorting. The non-dominated rankings are determined by the objective function value of the population.

Step 4: Congestion calculation. Measured by the distance between individuals and surrounding individuals: the greater the population congestion, the better the diversity.

Step 5: Crossover and mutation.

Step 6: The new offspring population, Q (n), is mixed with the parent population, P (n), to produce a new population, PQ (2n). The degrees of non-dominated sorting and crowding are calculated, and N excellent individuals are selected as the new parent population, P (n).

Step 7: Repeat the above steps until the evolutionary algebra set is reached.

**Figure 3:**
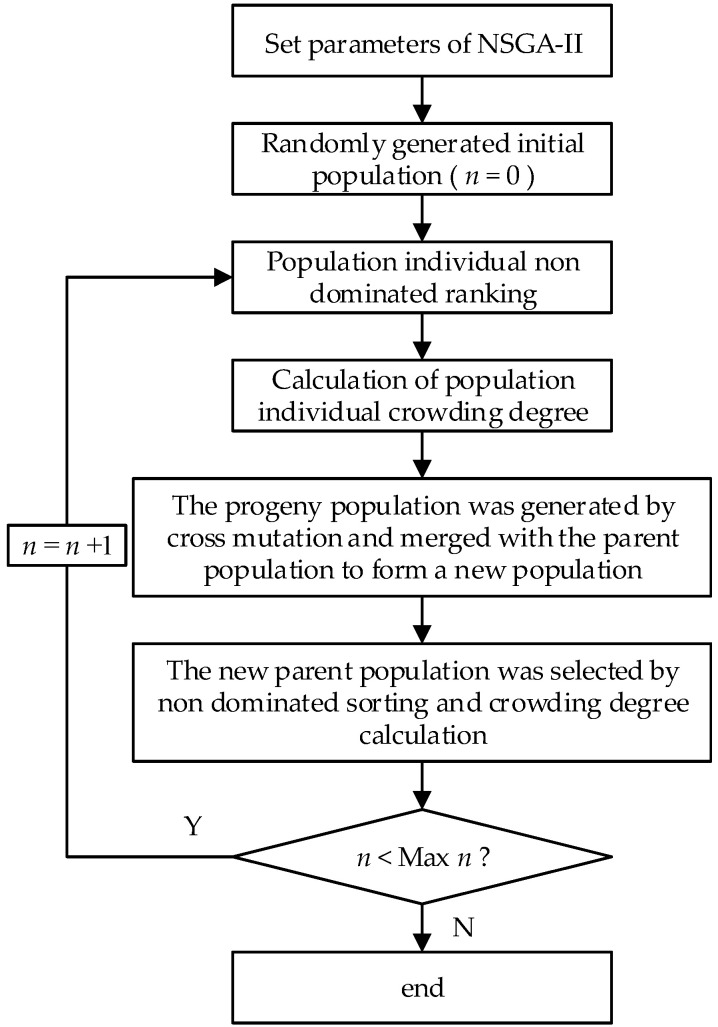
The flow chart of NSGA-II algorithm.

The flow chart of the NSGA-II algorithm used to solve the FPS-MOWAM model is shown in Figure 3.

#### 3.4.2. Scheme Optimisation Based on the Fuzzy Comprehensive Evaluation Model

The optimal solution set for the multi-objective model was obtained using the NSGA-II algorithm. In this study, a fuzzy comprehensive evaluation model was used to select the optimal configuration scheme. The evaluation index was standardised, as below, to eliminate the influence of its dimension and magnitude:

(14) $$r_{ij}=\frac{x_{ij}}{x_{i\max}-x_{i\min}},\begin{array}{cc} & x_{ij}\geq 0 \end{array}$$

(15) $$r_{ij}=\frac{x_{i\max}-x_{ij}}{x_{i\max}-x_{i\min}},\begin{array}{cc} & x_{ij}\geq 0 \end{array}$$

where $x_{ij}$ denotes the eigenvalues of scheme *j* for index *i*, $x_{i\max},x_{i\min}$ represent the maximum and minimum values of index *i* in all schemes respectively, and $r_{ij}$ represents the priority of scheme *j* for index *i*.

The weight vector of the two indices in the evaluation was set as $w=(w_{1},w_{2})$, and the value of programme *j* was as follows:

(16) $$u_{j}=\sum_{i=1}^{2}w_{i}\times r_{ij}$$

where $u_{j}$ represents the fuzzy evaluation value of scheme *j*. The maximum scheme of $u_{j}$ is the best scheme.

## 4. Results and Recommendations

### 4.1. Analysis of Wet–Dry Encounters in the Yellow River Basin

#### 4.1.1. Precipitation Spatial Clustering

**Figure 4:**
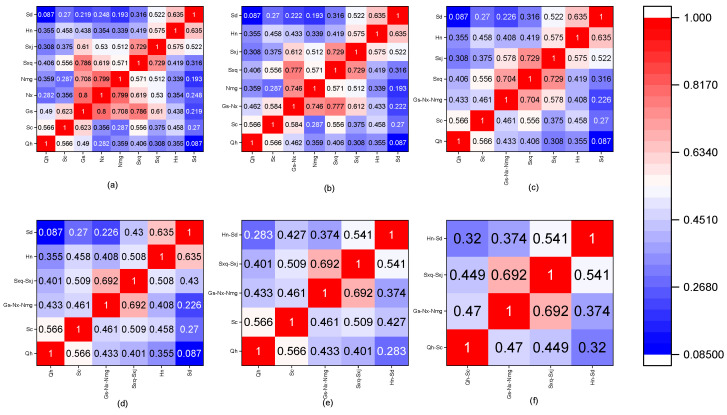
Correlation coefficient of precipitation among the nine provinces (**a**). Correlation coefficient of precipitation between Gs-Nx and other 7 provinces (**b**). Correlation coefficient of precipitation between Gs-Nx-Nmg and other 6 provinces (**c**). Correlation coefficient of precipitation among Gs-Nx-Nmg, Sxq-Sxj and other 4 provinces (**d**). Correlation coefficient of precipitation among Gs-Nx-Nmg, Sxq-Sxj, Hn-Sd and other 2 provinces (**e**). Correlation coefficient of precipitation among Qh-Sc, Gs-Nx-Nmg, Sxq-Sxj, and Hn-Sd (**f**).

The Spearman correlation and spatial cluster analyses were applied to precipitation data for the period 1960–2010 to generate a spatial cluster analysis of precipitation in nine provinces of the Yellow River Basin. The Spearman correlation coefficient is shown in Figure 4. Figure 4a–f shows the process of cluster merging according to precipitation correlation and the precipitation correlation coefficient of each region after merging. According to the strong correlation principle among provinces within a merged region, the nine provinces in the Yellow River Basin were merged into five regions: Qh, Sc, Gs-Nx-Nmg, Sxq-Sxj, and Hn-Sd. Furthermore, because the area of Sichuan Province within the Yellow River Basin accounts for only 2.1% of the total area of the latter, and given the historical perspective of water supply, the supply of Yellow River water to Sichuan Province accounts for only 0.1% of the total water supplied from the basin. Although the correlation with precipitation in other provinces is not strong, using allocation as an independent calculation unit in the overall allocation of water from the Yellow River Basin both increases the number of scenarios to be analysed and cannot reflect a more effective allocation of water resources in the basin. Therefore, based on the precipitation correlation, Sichuan Province and Qinghai Province are merged to form a new region, denoted Qh-Sc. The final four regions (Qh-Sc, Gs-Nx-Nmg, Sxq-Sxj, and Hn-Sd) were formed to analyse the wet–dry encounters in terms of the allocation of water resources in the Yellow River Basin.

#### 4.1.2. Joint Distribution Function Optimisation

The gamma, lnorm, norm, logis, and Weibull distributions commonly used in hydrometeorology were selected to fit the distribution functions of the Yellow River runoff and precipitation in the four study regions. The MAE (Mean Absolute Error), RMSE (Root Mean Square Error), and PPCC (Probability Plot Correlation Coefficient) values were calculated using the goodness-of-fit test method, and the results are shown in Table 1. The optimal distribution of runoff from the Yellow River is a gamma distribution, whereas the optimal distribution of precipitation in Qh-Sc, Gs-Nx-Nmg, Sxq-Sxj, and Hn-Sd is a Weibull distribution.

Four copula functions—Clayton, Gumbel, Frank, and Joe—were selected to fit the joint distribution functions of runoff and regional precipitation in the Yellow River Basin. The AIC (Akaike Information Criterion) and RMSE values of each of these functions were obtained using the calculations shown in Table 2. According to the principle that a small AIC and RMSE would result in a better fitting effect, the Frank copula function was selected to construct the joint distribution function for basin runoff and regional precipitation in the Yellow River Basin.

#### 4.1.3. Scenarios and Probabilities of Wet–Dry Encounters between the Yellow River Basin and Regions

**Figure 5:**
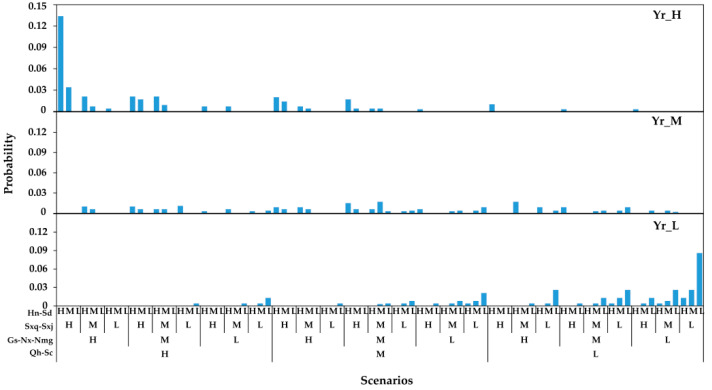
Scenarios and probabilities of wet–dry encounters in the Yellow River Basin and its regions.

**Figure 6:**
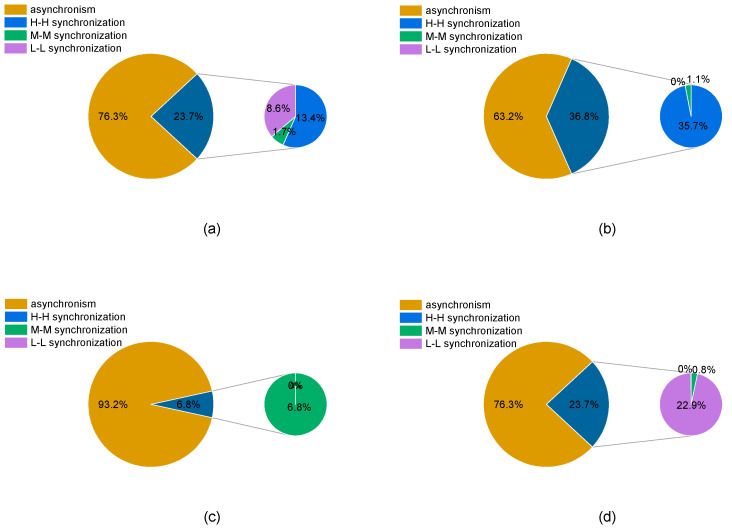
The synchronous and asynchronous probabilities of wet–dry encounters occurring in the Yellow River Basin and its regions (**a**), the synchronous and asynchronous probabilities of wet–dry encounters occurring in the regions under the high flow year in the Yellow River Basin (**b**), the synchronous and asynchronous probabilities of wet–dry encounters occurring in the regions under the medium flow year in the Yellow River Basin (**c**), the synchronous and asynchronous probabilities of wet–dry encounters occurring in the regions under the low flow year in the Yellow River Basin (**d**).

The scenarios and probabilities of wet–dry encounters in the Yellow River Basin were calculated based on the joint distribution functions of runoff and regional precipitation in the Yellow River Basin determined using the Frank copula function. The results are shown in Figure 5, and the synchronous and asynchronous results for wet–dry encounters between the basin and regions are shown in Figure 6. The synchronous and asynchronous probabilities in the Yellow River Basin were 0.237 and 0.763 respectively, showing that the asynchronous probability was 3.2 times higher than the synchronous probability, as shown in Figure 6a. The inconsistency phenomenon in the Yellow River was significant. There were also significant differences in the occurrence scenarios and probability of wet–dry encounters in various regions under the high, medium, and low flow conditions in the Yellow River Basin. When the Yellow River Basin was in a high flow year, the synchronous probability of the four regions was 0.368 and the asynchronous probability was 0.632, as shown in Figure 6b. When the basin was in a medium flow year, these figures decreased and increased respectively, to 0.068 and 0.932, as shown in Figure 6c. Finally, when the Yellow River Basin was in a low flow year, the synchronous and asynchronous probabilities for the four regions were 0.237 and 0.763 respectively, as shown in Figure 6d. There is a close relationship between the wet and dry areas in the Yellow River Basin and the upper and middle reaches. When the Yellow River Basin is in a high flow year, the provinces in the upper and middle reaches have a greater probability of encountering a wet year; however, the probability of this occurring in the lower reaches provinces is weak. However, when the Yellow River Basin is in a dry year, there is a high probability that the provinces in its upper and middle reaches will have dry years. Finally, when the Yellow River Basin is in a medium flow year, the wet–dry encounter scenarios and their probabilities are more uniform among all provinces.

This also shows that the traditional method of synchronous distribution of water resources is not applicable in the Yellow River Basin, and that the results of this method of allocation therefore do not accurately solve the contradiction between supply and demand in the basin; in fact, it could even aggravate this contradiction because the allocation scheme is unreasonable. Therefore, incorporating wet–dry encounters into water resource allocation and formulating related schemes based on these can better reflect the relationship between supply and demand for water resources in the basin. Using this method will also improve the adaptability of the allocation schemes.

### 4.2. Water Demand Forecast for Each Province in the Yellow River Basin

**Figure 7:**
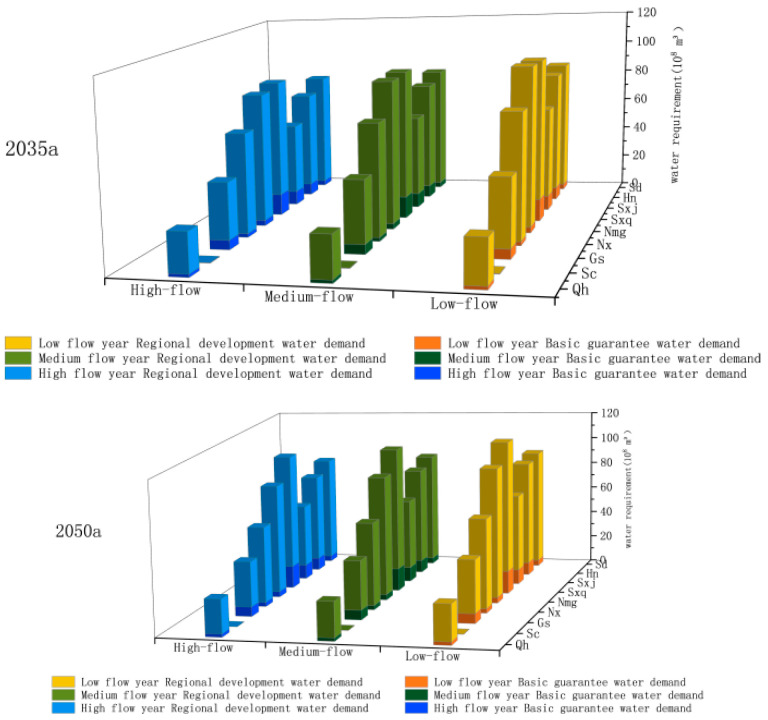
Forecast of water demand of each province in 2035a and 2050a.

**Figure 8:**
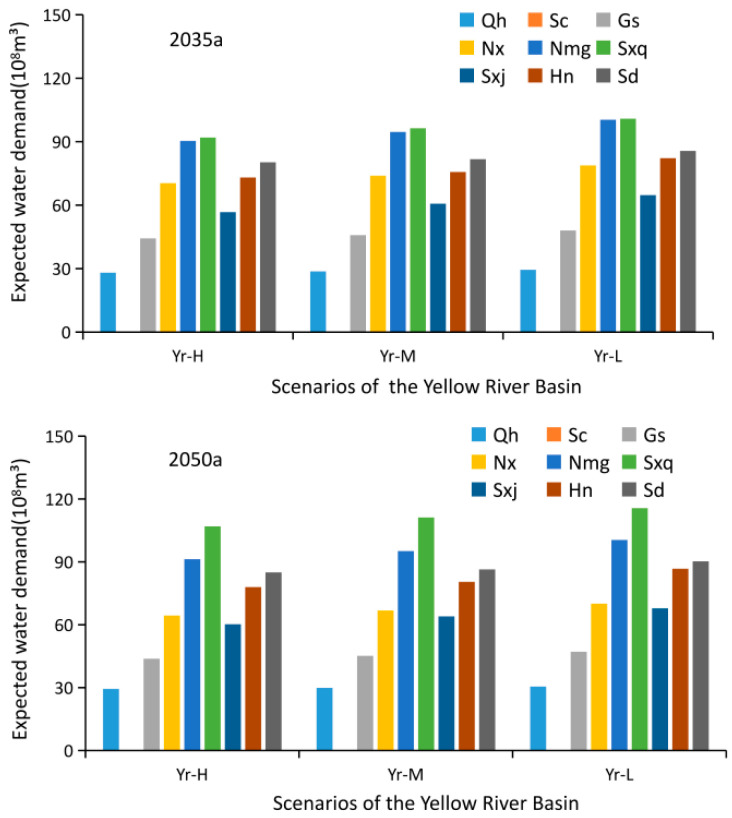
Expected water demand from each province in 2035a and 2050a.

Predicting the demand for water includes water needed for domestic purposes, regional development in each province, and basic ecosystem requirements in the main stream of the Yellow River. The results of water demand predictions under high, medium, and low flow conditions in each province in 2035 and 2050 are shown in Figure 7. These results, combined with various scenarios and probabilities that each would occur in every province under all flow conditions, produce the water demand expected from each province, as shown in Figure 8.

The basic ecosystem flow recorded by the main control stations on the Yellow River (Xiaheyan, Shizuishan, Toudaoguai, Tongguan, Gaocun, and Lijin) was used as the constraint condition for the ecosystem water demand in the river channel. The flow meets the water requirements demanded for ecological and sand washing purposes, as shown in Table 3. In addition, because the flow at Shizuishan station must reach 500 m^3^/s from December to March to meet ice prevention requirements for the main stream of the Yellow River, other months are constrained according to the requirements of the basic ecosystem water demand at Shizuishan station.

### 4.3. Water Resource Allocation Scheme in the Yellow River Basin

The NSGA-II algorithm used to solve FPS-MOWAM and the fuzzy comprehensive evaluation method used to determine the best allocation scheme were combined to obtain the water resource allocation scheme for the Yellow River Basin in 2035 and 2050, as shown in Table 4. Sichuan and Ningxia show a decreasing trend in water allocation over time. In the scenario in which the Yellow River goes through a low flow year, the trend in water allocation in Gansu decreases over time and that in other provinces increases. In terms of the increase and decrease, Shaanxi has the largest growth rate, exceeding 1.1 billion m^3^, and Ningxia has the largest decrease, exceeding 500 million m^3^. From the perspective of different wet–dry scenarios for the Yellow River, the amount of Yellow River water distributed in the low flow year exceeds that distributed in the medium flow year, whereas that distributed in the high flow year is the lowest. The main reason for this is that in low flow years, provinces are more likely to face dry scenarios. At these times, the water demand from each province increases; therefore, the amount of water allocated also increases. In a high flow year, the provinces are more likely to face a wet scenario, thereby reducing water demand and decreasing the distribution of water.

### 4.4. Analysis of Social and Economic Benefits

**Figure 9:**
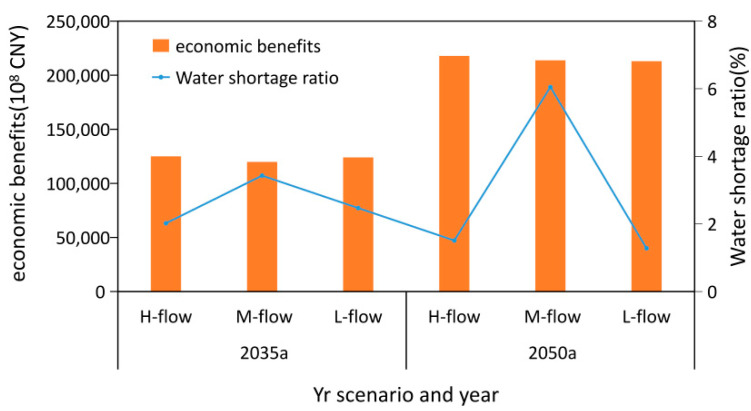
Expected water shortage rates and economic benefits in the Yellow River Basin under different water flow scenarios.

The water shortage rates and economic benefits expected in the Yellow River Basin under the optimal allocation scheme are shown in Figure 9. In 2035, the economic benefits expected in all scenarios in the high, medium, and low flow years were 12,499.6 billion CNY (Chinese Yuan), 11,982.7 billion CNY, and 12,399.0 billion CNY, respectively. The water shortage rates expected in all scenarios were 2.02%, 3.43%, and 2.47%, respectively. In 2050, the economic benefits expected in all scenarios in the high, medium, and low flow years were 21,781.3 billion CNY, 21,379.6 billion CNY, and 21,291.4 billion CNY respectively, while the water shortage rates expected in the same were 1.51%, 6.05%, and 1.28%, respectively. The water shortage rates expected in all scenarios show that high flow years are similar to low flow years, with the highest rate occurring in the medium flow year. This is because the probability of wet–dry encounters in each region of the Yellow River Basin is relatively concentrated in high and low flow years, and the wet–dry encounters in each region are relatively uniform in the medium flow year.

### 4.5. Analysis of the Difference between the Water Allocation Scheme and Expected Water Demand

**Figure 10:**
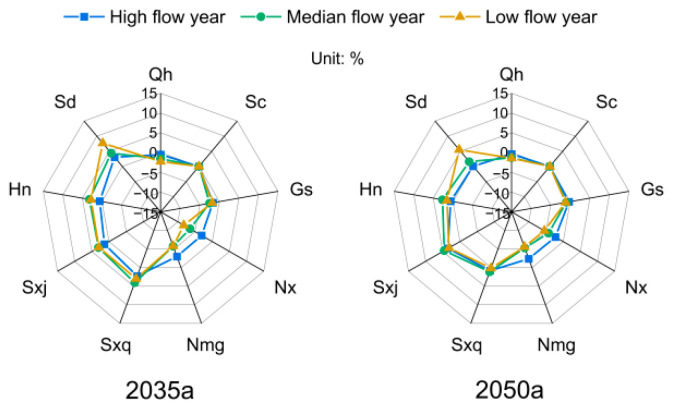
Difference rate between the water resource allocation scheme and water demand expected in the Yellow River Basin in 2035a and 2050a.

The expected water demand reflects the average water demand considering the full probability of wet–dry scenarios between basins and regions. Allocating water resources according to the expected water demand can ensure that the allocation scheme used can meet the multi-year average water demand. In the multi-objective water allocation scheme, the greater the resemblance of the water allocation scheme is to the expected water demand, the better it is from an equity perspective. Though, from an economic efficiency perspective, a more cost-effective scheme is preferred. The water allocation model needs to balance the economic efficiency objective with the equity objective. The difference rate between the water resource allocation scheme and the water demand expected in the Yellow River basin is shown in Figure 10. In 2035, the variation rate for each province ranges from −7.24% to 7.89%. Shandong Province’s allocation is greater than its expected water demand and has a variation rate of 7.89%. In 2050, the variation rate for each province ranges from −5.33% to 5.62%. Shandong’s allocation is greater than its expected water demand and has a variation rate of 5.62%. Ningxia’s water allocation is less than its expected water demand and has a variation rate of −5.33%. The spatial variation in 2035 shows that the water allocated to Shandong, Henan, Shanxi, and Shaanxi is greater than the expected water demand, the water allocated to Ningxia and Inner Mongolia is less than the expected water demand, and the water allocated to Qinghai, Sichuan, and Gansu is close to the expected water demand. In 2050, the water allocated to Shandong, Henan, and Shanxi is greater than the expected water demand, the water allocated to Ningxia and Inner Mongolia is less than the expected water demand, and the water allocated to Qinghai, Sichuan, Gansu, and Shaanxi is close to the expected water demand. The middle and lower reaches of the Yellow River Basin are more economically efficient per unit of water than the upper reaches and tend to allocate more water to more economically efficient areas in the multi-objective optimisation process to meet the water demand in a greater number of scenarios. Examining the Yellow River Basin’s wet and dry water years, the difference between provincial allocations and expected water demand is greatest in dry years and least in wet years. In a dry year, the provinces have more demand for water and the probability of a dry year increases. Therefore, in the pursuit of greater economic efficiency, water allocations are increasingly made to meet the needs of areas with high economic efficiency per unit of water, resulting in an increase in the difference between allocated water and expected water demand for each province in a dry year. Correspondingly, in a wet year, the water demand of each province decreases and the probability of a wet year increases. Thus, the water allocation can meet the water demand of each scenario more effectively, resulting in a smaller difference between the allocated water and the desired water demand.

### 4.6. Rationality Analysis of the Water Allocation Scheme Based on FPS-MOWAM

**Figure 11:**
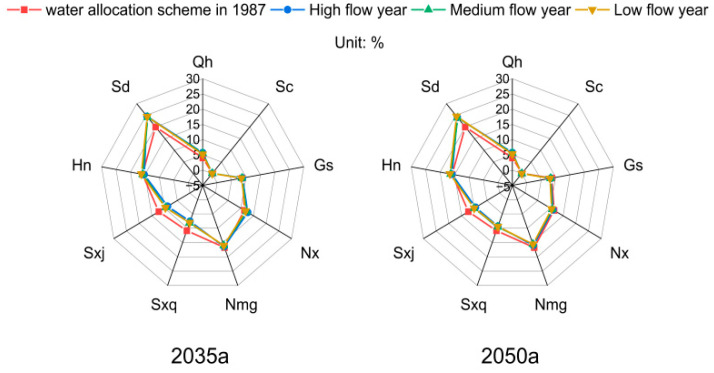
Consumption ratio of Yellow River surface water consumed under two water distribution schemes in 2035a and 2050a.

In terms of actual water demand, the differences in water supply between the FPS-MOWAM scheme and the 87WRAP scheme were analysed to verify that the configuration results were better adapted to the actual water demand. The difference in the distribution of the proportion of surface water consumed in each province can be used to compare the two distribution schemes, as shown in Figure 11. In 2035 and 2050, the proportions of surface water consumed in each province are similar under high, medium, and low flow conditions in the Yellow River basin; however, there is a certain difference between these values and those from the 87WRAP. Among them is the proportion of surface water consumed in Shandong, which was significantly higher in 2035 and 2050 than in the years used in 87WRAP; in Qinghai, Gansu, and Ningxia, the former was slightly higher than the latter. In Shaanxi and Shanxi, the values for the future years were lower than those in the 87WRAP, and those in Sichuan and Henan were relatively close. A main cause for this is that the 2035 and 2050 water demands of each province in the FPS-MOWAM model are based on actual water use data and social development data from 1996 to 2018, whereas the water demands in the 87WRAP model are based on what the water use data was at that time to project water use in 2000. However, the uneven development of the provinces over the last 40 years has led to changes in the pattern of water use, with Shandong using more water than was allocated under the 87WRAP model, while Shanxi and Shaanxi still have surplus water. Therefore, the conclusions of this study are closer to the actual situation than the 87WRAP.

**Figure 12:**
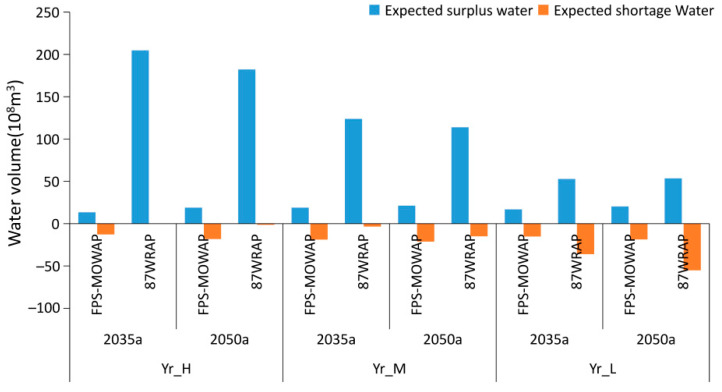
Expected surplus and shortage water under the FPS-MOWAM scheme and 87WRAP in 2035 and 2050 for the Yellow River Basin in wet, normal, and dry years.

In terms of the surplus and shortage water, the water allocation scheme rationality of the FPS-MOWAM scheme is analysed by verify the expected surplus and shortage water in each scenario. The expected water surplus and shortage of the Yellow River Basin in 2035 and 2050 in wet, normal, and dry years in the FPS-MOWAM scheme and 87WRAP are shown in Figure 12. In the FPS-MOWAM scheme, the expected surplus and shortage of water are relatively stable and within a small range. The expected water surplus is between 1.348 and 2.434 billion m^3^, and the expected water shortage is between −2.372 and −1.275 billion m^3^, showing an obvious symmetrical relationship. The regional water shortage problem can be solved by a complementary water supply between wet and dry regions. In the 87WRAP, there is a large surplus water in the Yr_H (the Yellow River in a high-flow year) and Yr_M (the Yellow River in a medium-flow year) scenarios, with the expected water surplus exceeding 10 billion m^3^, with a maximum of 20.474 billion m^3^ in the Yr_H scenario in 2035. However, the expected water shortage is in a smaller range. The variation in the expected water surplus and shortage was considerable. In the Yr_L (the Yellow River in a low-flow year) scenario, the expected water shortage increased rapidly, while the expected water surplus decreased rapidly. This indicates that the FPS-MOWAM scheme is better able to adapt to the water demand of the nine provinces along the Yellow River Basin under different wet–dry encounter scenarios, and the water allocation is more fitting.

## 5. Conclusions

This study proposed a multi-objective allocation model for basin water resources, considering all probability scenarios for wet–dry encounters (the FPS-MOWAM), and provided an effective solution for allocating basin water resources given conditions in which wet–dry encounters occur between the basin and regions. This method includes two core modules: an analysis of wet–dry encounters in terms of the basin and regions, based on a copula function, and a multi-objective-based optimal allocation of basin water resources considering all scenarios for wet–dry encounters. The model uses a copula function to construct a joint distribution function for basin runoff and regional precipitation that is then used to analyse the scenarios and probabilities of possible occurrences of basin–region wet–dry encounters. The FPS-MOWAM model is constructed using the minimum expected water shortage and the maximum expected economic benefit in each scenario as the goal. Considering the problem that there is a big difference in the scenarios and probabilities of wet–dry encounters under wet and dry conditions, the water resource allocation schemes for each region under the full probability scenario for wet and dry encounters were solved given high, medium, and low flow conditions in the basin, and the water resource allocation scheme was thus further refined.

The model was applied to the Yellow River Basin in China and the wet–dry encounters between the basin and nine provinces were analysed. The water distribution schemes for nine provinces under high, medium, and low flow conditions in the Yellow River Basin in 2035 and 2050 were calculated, and the results showed the following. (1) There is an obvious inconsistency among the high, medium, and low years in the Yellow River Basin, with an asynchronous probability of 0.763. In addition, in the wet and dry years in the Yellow River Basin, the wet and dry conditions therein are more closely related to wet and dry conditions in the upper and middle reaches of the river. In a medium flow year for the basin, each encounter becomes more scattered. (2) Calculations made using the model show that the economic benefits expected in each scenario in 2035 and 2050 increased from approximately 12 trillion to approximately 21 trillion, while the water shortage rate expected in each scenario under wet and dry conditions was controlled below 3%, and in normal years below 7%. (3) Comparing the allocation results with the water demand expected and the water diversion scheme currently in use showed that the difference rate between the allocation scheme and the water demand expected in each province in 2035 and 2050 was within ±10%. There was a certain difference between the allocation scheme based on the FPS-MOWAM model (proposed in this study) and the 87WRAP scheme (currently in use); however, the difference in the proportion of each province was within 5%.

## Figures and Tables

**Table 1:** Distribution functions for the goodness-of-fit test results.

Test Method	Distribution Function	Yr	Qh-Sc	Gs-Nx-Nmg	Sxq-Sxj	Hn-Sd
MAE	gamma	0.019072	0.054294	0.07684	0.083501	0.090433
	lnorm	0.021876	0.053756	0.07678	0.083491	0.090256
	norm	0.026867	0.055502	0.077037	0.083603	0.09093
	logis	0.02333	0.055292	0.077721	0.085438	0.093036
	Weibull	0.02989	0.051729	0.071843	0.077436	0.084873
RMSE	gamma	0.000408	0.002878	0.004356	0.004509	0.004715
	lnorm	0.000533	0.002913	0.004433	0.004583	0.004776
	norm	0.000811	0.002837	0.004221	0.004385	0.004619
	logis	0.000632	0.00303	0.004512	0.004739	0.004992
	Weibull	0.000993	0.002302	0.003503	0.003635	0.003877
PPCC	gamma	0.001914	0.001147	0.003778	0.00488	0.008173
	lnorm	0.002460	0.001196	0.003764	0.004882	0.008183
	norm	0.002031	0.001027	0.003796	0.004867	0.008147
	logis	0.002226	0.001175	0.003801	0.004906	0.008178
	Weibull	0.002367	0.000899	0.003836	0.004838	0.008118

**Table 2:** Goodness-of-fit test results for the copula functions.

Copula Function	AIC	RMSE
Clayton	−198.27	0.0581
Gumbel	−207.15	0.0459
Frank	−217.84	0.0435
Joe	−201.43	0.049

**Table 3:** Basic flow required for ecological purposes at the main control stations in the Yellow River Basin.

Station	Xiaheyan	Shizuishan	Toudaoguai	Tongguan	Huayuankou	Gaocun	Linjin
Runoff(m³/s)	200	150	50	50	150	120	50

**Table 4:** Water resource allocation scheme for the Yellow River Basin in 2035a and 2050a. Unit: 10^8^ m^3^.

Region	Groundwater	Transfer Water	Surface Water in the Yellow River Basin					
			2035a			2050a		
			H-Flow	M-Flow	L-Flow	H-Flow	M-Flow	L-Flow
Qh	3.27	0	24.65	24.98	25.52	26.04	26.23	26.79
Sc	0.01	0	0.31	0.31	0.31	0.12	0.13	0.13
Gs	5.68	0	37.86	39.00	41.46	37.97	39.04	40.90
Nx	7.68	0	60.52	61.47	64.58	55.25	56.26	58.58
Nmg	25.08	0	62.58	64.09	69.47	64.02	65.03	69.80
Sxq	29.51	16.37	48.17	54.36	58.00	61.97	66.59	69.98
Sxj	21.06	0	36.43	41.48	45.58	41.32	45.86	48.96
Hn	21.55	0	51.94	56.56	62.93	56.89	61.06	66.49
Sd	11.44	1.26	69.98	72.58	79.52	72.35	75.06	82.52
Total	125.28	17.63	392.44	414.83	447.37	415.94	435.26	464.16

## Data Availability

Not applicable.
